# Monocaprin, Monolaurin, and Monomyristin Eradicate *Staphylococcus aureus* Persister Cells Through Membrane Disruption

**DOI:** 10.3390/ph19050690

**Published:** 2026-04-28

**Authors:** Dae-Yoon Kim, Tae-Jong Kim

**Affiliations:** Department of Forest Products and Biotechnology, Kookmin University, Seoul 02707, Republic of Korea; dae111500@kookmin.ac.kr

**Keywords:** antibiotic tolerance, membrane disruption, monoglycerides, monolaurin, persister cells, *Staphylococcus aureus*

## Abstract

**Background/Objectives**: *Staphylococcus aureus* persister cells significantly undermine antimicrobial therapy through their transient antibiotic tolerance, contributing to chronic and recurrent infections. Although monoglycerides have shown potential as membrane-active antimicrobial agents, their effect on persister cells remains insufficiently understood. **Methods**: In this study, we evaluated the anti-persister activities of monocaprin, monolaurin, and monomyristin against *S. aureus* persister cells. Mechanistic analyses were performed using membrane permeability assays and fluorescence microscopy. **Results**: All three monoglycerides reduced persister cell survival, with varying degrees depending on fatty acid chain length. Monolaurin exhibited the greatest anti-persister activity, whereas monocaprin and monomyristin exerted concentration-dependent bactericidal effects. Mechanistic analyses revealed that these compounds increased membrane permeability, thereby compromising cell viability in *S. aureus* persister cells. In contrast, Tween 80 attenuated both the bactericidal effect and the increase in membrane permeability, supporting the involvement of membrane disruption in their mode of action. **Conclusions**: The antibacterial activity of monocaprin, monolaurin, and monomyristin against *S. aureus* is closely associated with membrane damage. These membrane-active monoglycerides represent promising antimicrobial candidates for the eradication of *S. aureus* persister cells.

## 1. Introduction

*Staphylococcus aureus* is a major human pathogen responsible for both acute and chronic infections [1]. Besides antimicrobial resistance, the persistence of *S. aureus* infection is often linked to the formation of persister cells, a subpopulation that exhibits transient tolerance to antibiotic treatment without developing genetic resistance [2,3]. Because persister cells can repopulate after therapy, they are one of the contributors to treatment failure and recurrent infection [2,3].

Conventional antibiotics are often less effective against persister cells owing to their reduced metabolisms, which results in reduced susceptibility to growth-dependent antibacterial mechanisms [3,4]. This therapeutic challenge has driven increasing interest in agents that target alternative pathways, particularly the bacterial membrane [5]. Membrane-active compounds are particularly relevant because they may retain bactericidal activity against dormant or slow-growing cells, thereby providing a potential strategy for persister eradication [5,6]. A recent investigation has also demonstrated that suppression of *S. aureus* persister cells can be achieved by modulating membrane-associated physiology, including altered membrane fluidity, thereby supporting the broader relevance of membrane-targeted anti-persister strategies [7].

Monoglycerides are membrane-active antimicrobial lipids that exert bactericidal activity against Gram-positive bacteria, including *S. aureus* [8,9]. Saturated monoglycerides such as monocaprin, monolaurin, and monomyristin may disrupt bacterial membranes and thereby impair cell viability [9,10]. However, their effects on *S. aureus* persister cells have not been fully elucidated. In particular, experimental evidence linking their anti-persister activity to membrane disruption remains limited.

Thus, we evaluated the anti-persister activities of monocaprin, monolaurin, and monomyristin against *S. aureus*. We then examined their effects on membrane permeability to determine the association between membrane damage and elimination of persister cells. This study demonstrated the antimicrobial activity of these monoglycerides against *S. aureus* persister cells and established membrane disruption as a key mechanism underlying their activity.

## 2. Results

### 2.1. Anti-Persister Activities of Monocaprin, Monolaurin, and Monomyristin Against S. aureus

To evaluate the effects of monoacylglycerols on persister cells, experimental conditions that selectively enrich this subpopulation should be established. Persister cells can be isolated by exposing bacterial cultures to antibiotic concentrations significantly higher than the minimum inhibitory concentration (MIC), which effectively eliminates metabolically active cells. Under these high antibiotic concentrations, only a small fraction of dormant or metabolically less active cells—defined as persister cells—survive, allowing their subsequent analysis. Therefore, the MIC values of each monoacylglycerol were first determined to establish appropriate conditions for persister cell assays (Table 1).

**Figure 1:**
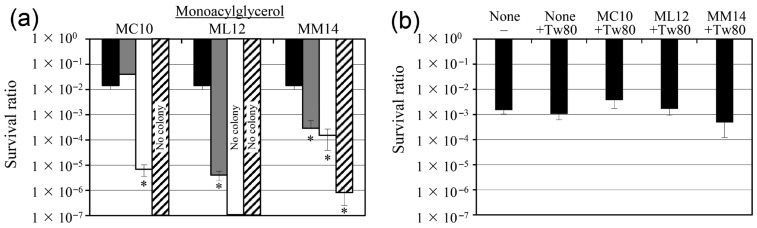
Bactericidal activities of monocaprin, monolaurin, and monomyristin against *Staphylococcus aureus* persister cells and neutralization of their activities by Tween 80. (**a**) Survival of *S. aureus* persister cells after treatment with monocaprin (MC10), monolaurin (ML12), or monomyristin (MM14) at 0 mM (black bars), 0.1 mM (gray bars), 1 mM (open bars), and 10 mM (diagonal bars) with 2.5 mg/L oxacillin. Persister cell counts were quantified (CFUs) after 24 h of treatment. (**b**) Effect of 1% Tween 80 (Tw80) on the bactericidal activities of monocaprin, monolaurin, and monomyristin. Persister cell survival was determined after co-treatment with each monoglyceride solution containing 1 mM, 2.5 mg/L oxacillin, and 1% Tween 80. Dimethyl sulfoxide (1%) was used as the control. The survival ratio was calculated as the number of viable cells after 24 h of treatment divided by the number of viable cells before treatment. Data are expressed as the mean ± standard deviation (SD) of three independent experiments (* *p* < 0.05). “No colony” indicates that no viable cells were detected on tryptic soy agar (TSA) plates. “None” indicates treatment with 2.5 mg/L oxacillin alone without any monoacylglycerol.

The anti-persister activities of monocaprin, monolaurin, and monomyristin against *S. aureus* were evaluated by quantifying bacterial counts (colony-forming units [CFUs]) following oxacillin treatment. All three monoglycerides reduced the number of *S. aureus* persister cells in a concentration-dependent manner (Figure 1a).

Monolaurin exhibited the strongest antimicrobial activity, eliminating all detectable persister cells at concentrations ≥ 1 mM. Monomyristin also significantly reduced persister survival at 1 mM and completely eradicated detectable cells at 10 mM. In contrast, monocaprin showed the weakest activity and required 10 mM to achieve complete elimination of persister cells. Under these conditions, the surviving population is considered to represent persister cells rather than resistant mutants, as previously defined [11,12].

### 2.2. Tween 80 Neutralizes the Antimicrobial and Membrane-Permeabilizing Effects of Monoglycerides

To examine the association between the bactericidal activity of the tested monoglycerides and membrane disruption, their effects were evaluated in the presence of Tween 80. Tween 80 markedly attenuated the bactericidal activities of monocaprin, monolaurin, and monomyristin (Figure 1b). Although these compounds substantially reduced the number of surviving persister cells, their bactericidal effects were largely eliminated with the addition of Tween 80.

**Figure 2:**
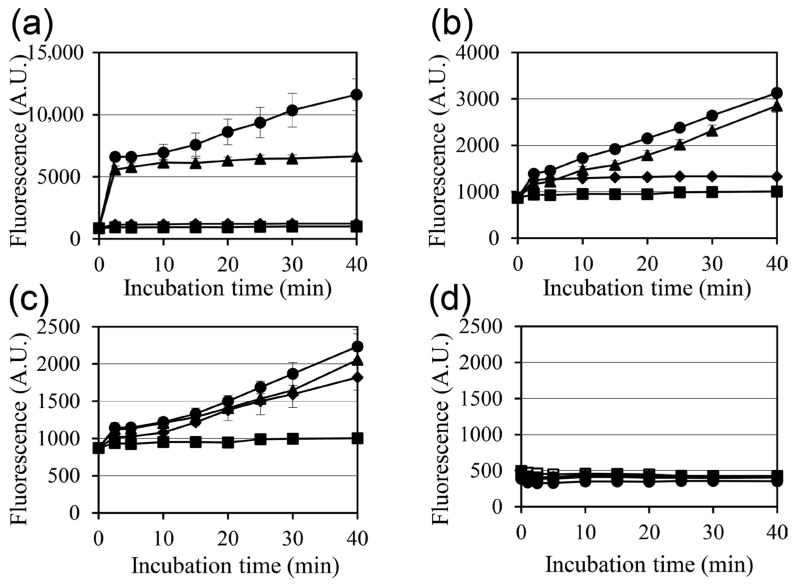
Membrane permeability of *Staphylococcus aureus* following treatment with monoacylglycerols. (**a**–**c**) SYTOX™ Green uptake in cells treated with monocaprin (**a**), monolaurin (**b**), or monomyristin (**c**) at 0 mM (■), 0.1 mM (◆), 1 mM (▲), or 10 mM (●). (**d**) Effect of 1% Tween 80 on monoglyceride-induced membrane permeabilization. The tested conditions were no treatment (◻), Tween 80 alone (■), 1 mM monocaprin with Tween 80 (◆), 1 mM monolaurin with Tween 80 (▲), and 1 mM monomyristin with Tween 80 (●). Dimethyl sulfoxide (1%) was used as the control. Data are presented as mean ± SD (*n* = 3).

**Figure 3:**
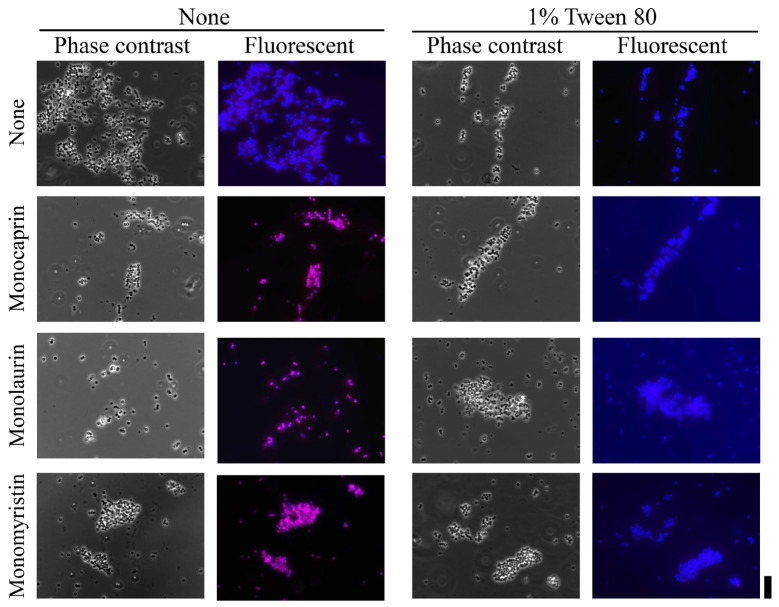
Fluorescence microscopic analysis of membrane damage in *Staphylococcus aureus* cells treated with monoglycerides, namely 1 mM monocaprin, 1 mM monolaurin, or 1 mM monomyristin, in the absence (the left two columns) or presence of 1% Tween 80 (the right two columns) and stained with propidium iodide (PI, pink) and 4′,6-diamidino-2-phenylindole dihydrochloride (DAPI, blue). Positive PI staining indicates membrane damage and loss of viability, whereas DAPI visualizes all cells regardless of viability. Phase-contrast images show the cell morphology. Fluorescence images are merged images of PI and DAPI signals. Images were acquired at 1000× magnification (scale bar = 10 μm).

A similar trend was observed in the membrane permeability assay. As shown in Figure 2d, Tween 80 reduced the membrane-permeabilizing effects of all three monoglycerides to levels that were comparable with those of the untreated control (Figure 2). Fluorescence microscopy provided further confirmation, showing that the strong propidium iodide (PI)-positive staining induced by monoglyceride treatment was substantially suppressed in the presence of Tween 80 (Figure 3). Overall, the findings indicate that Tween 80 neutralizes both the bactericidal and membrane-permeabilizing effects of monoglycerides, suggesting an association between persister cell elimination and membrane damage.

### 2.3. Membrane-Damaging Effects of Monoglycerides on S. aureus

The membrane-damaging effects of monocaprin, monolaurin, and monomyristin on *S. aureus* were assessed using SYTOX Green uptake assays and fluorescence microscopy. Treatment with all three monoglycerides increased membrane permeability in a concentration-dependent manner compared with the untreated control, indicating disruption of membrane integrity (Figure 2a–c).

Among the three monoglycerides, monolaurin induced the greatest increase in membrane permeability in a dose-dependent manner, with a marked effect observed even at low concentrations. In contrast, monocaprin showed the weakest effect, requiring higher concentrations to achieve comparable permeabilization. Fluorescence microscopy further confirmed that monoglyceride-treated cells exhibited significantly greater PI staining than untreated cells, indicating membrane damage and loss of viability (Figure 3). Collectively, these results demonstrate that monocaprin, monolaurin, and monomyristin disrupt membrane integrity and thereby exert their antibacterial effects against *S. aureus* persister cells.

## 3. Discussion

In this study, monocaprin, monolaurin, and monomyristin all exhibited antibacterial activity against *S. aureus* persister cells with varying potencies. Monolaurin showed the strongest effect, eradicating detectable persister cells at lower concentrations than monocaprin or monomyristin. These differences were consistent with the membrane permeability assays, in which monolaurin also produced the greatest increase in membrane permeability. Collectively, these findings demonstrate that membrane disruption is a central mechanism underlying the bactericidal activity of these monoglycerides [8,13].

The neutralizing effect of Tween 80 further strengthens this interpretation. Tween 80 markedly attenuated the bactericidal activity against surviving cells and membrane-permeabilizing effects of all three monoglycerides, indicating that their antibacterial activity is largely dependent on membrane interactions. Importantly, Tween 80 alone did not significantly change persister cell survival under the experimental conditions, as evidenced by the comparable survival ratios in the oxacillin-only and oxacillin-plus-Tween 80 conditions (Figure 1b). This result suggests that Tween 80 does not exert a measurable direct effect on persister viability in this assay. Therefore, the observed reduction in antibacterial and membrane-permeabilizing effects in the presence of Tween 80 is more likely attributable to interference with monoacylglycerol–membrane interactions, rather than to a direct effect of Tween 80 on cell viability. A similar relationship between reduced persister cell survival, increased membrane permeability, and Tween 80–mediated neutralization has also been reported in *S. aureus* treated with Coicis Semen–derived active components [14]. This observation is consistent with the established surfactant-like behavior of monoglycerides, which can insert into lipid bilayers and destabilize membrane structures [8]. Because persister cells are tolerant to many conventional antibiotics owing to their altered physiological state, membrane-active compounds such as monoglycerides may represent an effective strategy for killing cells that are less susceptible to growth-dependent antibacterial mechanisms [5,6].

The differences in activity among the tested monoglycerides also provide insights into their structure-related antibacterial properties. The superior potency of monolaurin in both bactericidal activity and membrane permeabilization in *S. aureus* cells may be associated with the length of its fatty acid chain. In contrast, monocaprin showed weaker activity and required higher concentrations to achieve comparable effects, whereas monomyristin exhibited intermediate activity. Notably, previous studies on medium-chain fatty acids have shown that octanoic acid, decanoic acid, and lauric acid eliminated *S. aureus* persister cells, whereas myristic acid showed no detectable activity within the tested concentration range [13]. In contrast, monomyristin showed detectable activity and induced marked membrane permeabilization in the present study, indicating that the addition of a glycerol moiety may enhance membrane interaction and thereby improve antimicrobial efficacy [8].

This study has several limitations. First, membrane permeability was measured in vegetative cells rather than in persister cells. Because vegetative cells are metabolically active, they may respond to membrane stress differently from persister cells, which exhibit markedly reduced metabolic activity [4,6]. This difference may partly explain the apparent discrepancy between bactericidal activity and membrane permeability at lower concentrations. For example, while monolaurin exhibited notable bactericidal activity at 0.1 mM, membrane permeabilization in vegetative cells remained limited. Therefore, although our findings strongly support an association between membrane damage and bactericidal activity, the membrane effects observed here may not fully reflect the physiological state of persister cells. Second, this study was conducted using a single laboratory strain, *S. aureus* ATCC 6538. Because persister formation and membrane properties may vary among strains, including clinically relevant methicillin-resistant isolates, the generalizability of our findings remains to be established. Third, persister cells were generated using an oxacillin-based in vitro model. Although this model enables reproducible evaluation of anti-persister activity, it may not fully represent the heterogeneous physiological states of persister cells formed under other clinically relevant conditions, such as nutrient limitation, stationary phase, or biofilm-associated growth. Fourth, the mechanistic evidence presented here is largely based on membrane permeability assays, fluorescence microscopy, and Tween 80–mediated neutralization. Although these data strongly support membrane involvement, they do not fully define the precise biophysical events underlying killing, such as membrane depolarization, leakage of intracellular contents, or ultrastructural disruption. Finally, the clinical applicability of these findings remains limited by the in vivo nature of the experiments, which did not include biofilm models, host–cell toxicity assays, or in vivo infection models. Further studies addressing these aspects will be necessary to determine the therapeutic potential and safety window of these monoglycerides.

Monoacylglycerols, including monocaprin, monolaurin, and monomyristin, are structurally related to lipid intermediates naturally generated during human fat digestion and absorption. During the digestion of dietary triglycerides, pancreatic lipases preferentially hydrolyze the *sn*-1 and *sn*-3 positions, yielding 2-monoacylglycerols (2-MAGs), which are subsequently absorbed by intestinal epithelial cells [15,16]. However, the monoacylglycerols used in this study are in the 1-monoacylglycerol (1-MAG) form, whereas physiological lipid digestion mainly produces 2-MAGs. These positional isomers differ in their biochemical properties: 2-MAGs are the preferred substrates in intestinal lipid re-esterification pathways, while 1-MAGs may undergo acyl migration and display different metabolic stability and membrane interactions [17,18,19]. Moreover, monoacylglycerols have been widely recognized as biologically active lipids with antimicrobial and membrane-interacting properties [20,21,22]. However, despite their physiological relevance, quantitative data describing the endogenous levels of these specific monoacylglycerols in human plasma or tissues remain limited. Therefore, their potential effects on human cells, particularly at concentrations required for antimicrobial activity, cannot be reliably predicted with certainty. Further studies evaluating cytotoxicity, cellular responses, and the therapeutic windows in mammalian systems are necessary to assess their clinical applicability. This consideration is particularly important for membrane-active compounds, as their potential for non-selective interactions with host–cell membranes may raise safety concerns [23,24,25].

Overall, this study demonstrates that membrane-active monoglycerides, particularly monolaurin, are promising agents for controlling persistent *S. aureus* infections. Their ability to disrupt bacterial membrane integrity may facilitate targeting antibiotic-tolerant persister cells and support combination approaches with conventional antibiotics [5,6]. Further studies are warranted to examine the activity of these monoglycerides in biofilm-associated systems and in vivo infection models and to more precisely define the influence of structural differences among monoglycerides on membrane interaction and antimicrobial efficacy.

## 4. Materials and Methods

### 4.1. Strain, Chemicals, and Cultivation Conditions

*S. aureus* ATCC 6538 was obtained from the Korean Collection for Type Cultures (Korean Research Institute of Bioscience and Biotechnology, Jeongeup, Republic of Korea) and stored at −80 °C in 25% glycerol. This strain is a well-characterized laboratory strain that lacks known antibiotic resistance traits and is widely used for antimicrobial susceptibility testing. The MICs of monocaprin, monolaurin, and monomyristin against *S. aureus* ATCC 6538 were determined and have been summarized in Table 1.

Oxacillin sodium salt (CN: sc-224180B) was purchased from Santa Cruz Biotechnology Inc. (Dallas, TX, USA) and dissolved in triple-distilled water. Monocaprin (CN: M1072), monolaurin (CN: G0081), and monomyristin (CN: M1073) were purchased from Tokyo Chemical Industry Co., Ltd. (Tokyo, Japan). Dimethyl sulfoxide (DMSO; CN: 000D0458; Samchun Chemical Co., Ltd., Seoul, Republic of Korea) was used as a solvent. SYTOX™ Green Nucleic Acid Stain (CN: S7020) was purchased from Thermo Fisher Scientific Korea Ltd. (Seoul, Republic of Korea). Saline solution was prepared with triple-distilled water to a final concentration of 0.85% (*w*/*v*) sodium chloride. Bacterial cells were cultured in tryptic soy broth (TSB; CN: 211825; Becton Dickinson Korea Co., Ltd., Seoul, Republic of Korea) and on TSA, the latter of which was prepared by supplementing TSB with 1.5% Bacto Agar (CN: 214010; Becton Dickinson Korea Co., Ltd.). For preculture, cells were streaked onto TSA and incubated at 37 °C for 24 h. A single colony was subsequently inoculated into 5 mL TSB and cultured at 37 °C with shaking at 250 rpm for 24 h.

### 4.2. Determination of Minimum Inhibitory Concentration

The MICs of monocaprin, monolaurin, and monomyristin against *S. aureus* were determined using a standard broth microdilution assay [26]. *S. aureus* was precultured in 5 mL TSB at 37 °C with shaking at 250 rpm for 24 h. The culture was then diluted to an optical density at 600 nm (OD_600_) of 0.05 in fresh TSB. Each monoglyceride was serially diluted twofold in TSB, and the diluted bacterial suspension was added to achieve a final volume of 5 mL. The cultures were incubated at 37 °C for 24 h. The lowest treatment concentration that completely inhibited visible bacterial growth was recorded as the MIC.

### 4.3. Persister Cell Assay

To quantify persister cell counts, *S. aureus* was precultured under the same experimental conditions described above. The culture was diluted to an OD_600_ of 0.05 in 4.5 mL fresh TSB and incubated at 37 °C with shaking at 250 rpm for 3 h. Oxacillin was then added at 10× the MIC (2.5 mg/L), and the cultures were incubated for an additional 24 h. For the treatment groups, each monoglyceride, either alone or in combination with 1% Tween 80, was added simultaneously with oxacillin. DMSO (1%) solution was used as the control. Samples were collected before and after 24 h of antibiotic treatment. Cells were washed by centrifugation at 6800× *g* for 1 min, resuspended in saline, diluted, and inoculated onto TSA plates. After incubation at 37 °C for 24 h, CFUs were counted. The survival ratio was calculated as the number of CFUs after 24 h of treatment divided by the number of CFUs before treatment. All experiments were performed in triplicate.

### 4.4. Fluorescence Microscopy

Fluorescence microscopy was performed to assess changes in membrane permeability induced by monoglyceride treatment. PI and 4′,6-diamidino-2-phenylindole dihydrochloride (DAPI) were used to distinguish membrane-compromised and total cells, respectively. Using the same preculture and inoculation conditions as those used in the persister cell assay, cultures were incubated for 3 h, washed with saline by centrifugation at 6800× *g* at 4 °C for 5 min, and treated with monoglycerides or monoglycerides plus 1% Tween 80 for 30 min at 25 °C. PI and DAPI were then added to final concentrations of 50 µg/mL each, and the cells were incubated for an additional 5 min. Images were acquired using a Zeiss Scope A1 microscope (AXIO, Carl Zeiss Co. Ltd., Seoul, Republic of Korea). PI fluorescence was observed at an excitation wavelength of 573 nm and emission wavelength of 591 nm. DAPI fluorescence was observed at 358 nm excitation and 461 nm emission. Images were analyzed using ZEN lite (Carl Zeiss Co. Ltd.) and Fiji software (version 2.15.1) [27].

### 4.5. Membrane Permeability Measurement

Membrane permeability was measured using SYTOX™ Green Nucleic Acid Stain. After preculture under the same conditions used for the persister cell assay, cells were washed with saline by centrifugation at 6800× *g* at 4 °C for 5 min and then incubated with SYTOX™ Green at a final concentration of 2.5 µM for 10 min. Monoglycerides were added to the treatment groups, either alone or together with 1% Tween 80. DMSO (1%) was used as the control. Fluorescence was measured in a black 96-well plate (CN: 30296; SPL Life Sciences Co., Ltd., Pocheon, Republic of Korea) using a Synergy™ LX Multi-Mode Reader (BioTek Instruments Korea Ltd., Seoul, Republic of Korea) with excitation at 485 nm and emission at 528 nm. All experiments were performed in triplicate.

### 4.6. Statistical Analysis

All experiments were performed in triplicate, and the data are expressed as mean ± standard deviation. Statistical significance was determined using Student’s *t*-test, with statistical significance set at a *p*-value < 0.05.

## 5. Conclusions

This study demonstrated the antimicrobial activity of monocaprin, monolaurin, and monomyristin against *S. aureus* persister cells, which is closely associated with membrane damage. Monolaurin showed the greatest potency, reducing detectable persister cells to lower levels than those achieved with monocaprin and monomyristin. The significant activity exhibited by monomyristin indicates that the linkage of myristic acid to glycerol may enhance membrane interactions and improve anti-persister efficacy. The neutralizing effect of Tween 80, together with the observed increase in membrane permeability, supports membrane disruption as a major factor underlying the antibacterial action of these compounds. Overall, our findings highlight membrane-active monoglycerides, particularly monolaurin, as promising candidates for controlling persistent *S. aureus* infections. Further evaluations of combination therapy and infection models are warranted to validate their therapeutic potential.

## Figures and Tables

**Table 1:** Minimum inhibitory concentrations (MICs) of monoacylglycerols against *Staphylococcus aureus* ATCC 6538.

Monoacylglycerol	Monocaprin	Monolaurin	Monomyristin
MIC	1 mM (0.246 g/L)	0.5 mM (0.137 g/L)	0.5 mM (0.151 g/L)

## Data Availability

The original contributions presented in this study are included in the article. Further inquiries can be directed to the corresponding author.

## Abbreviations

The following abbreviations are used in this manuscript:

CFUs	Colony-forming units
CN	Catalog number
DAPI	4′,6-Diamidino-2-phenylindole dihydrochloride
DMSO	Dimethyl sulfoxide
MAG	Monoacylglycerol
MC10	Monocaprin
MICs	Minimum inhibitory concentrations
ML12	Monolaurin
MM14	Monomyristin
PI	Propidium iodide
TSA	Tryptic soy agar
TSB	Tryptic soy broth
Tw80	Tween 80
